# Video Compression for Screen Recorded Sequences Following Eye Movements

**DOI:** 10.1007/s11265-021-01719-2

**Published:** 2021-11-23

**Authors:** Diego Jesus Serrano-Carrasco, Antonio Jesus Diaz-Honrubia, Pedro Cuenca

**Affiliations:** 1 Grupo de Redes y Arquitecturas de Altas Prestaciones, Universidad de Castilla-La Mancha, Castilla-La Mancha, Spain; 2 E.T.S. de Ingenieros Informaticos, Centro de Tecnología Biomédica, Universidad Politécnica de Madrid, Madrid, Spain

## Abstract

With the advent of smartphones and tablets, video traffic on the Internet has increased enormously. With this in mind, in 2013 the *High Efficiency Video Coding* (HEVC) standard was released with the aim of reducing the bit rate (at the same quality) by 50% with respect to its predecessor. However, new contents with greater resolutions and requirements appear every day, making it necessary to further reduce the bit rate. Perceptual video coding has recently been recognized as a promising approach to achieving high-performance video compression and eye tracking data can be used to create and verify these models. In this paper, we present a new algorithm for the bit rate reduction of screen recorded sequences based on the visual perception of videos. An eye tracking system is used during the recording to locate the fixation point of the viewer. Then, the area around that point is encoded with the base *quantization parameter* (QP) value, which increases when moving away from it. The results show that up to 31.3% of the bit rate may be saved when compared with the original HEVC-encoded sequence, without a significant impact on the perceived quality.

## Introduction

We live in a digital society in which the consumption of multimedia content is constantly increasing. For instance, in 2017 video traffic amounted to 75% of all Internet traffic and it is expected to rise up to 82% by 2022 [1]. This is mainly due to advances in transmission and compression technologies. However, as a result, users expect higher standards in terms of quality, video resolution, frames per second, and so on.

Taking this fact into consideration, and with the aim of achieving a greater bit rate reduction while preserving quality, the *Joint Collaborative Team on Video Coding* (JCT-VC) finished the first version of the *High Efficiency Video Coding* (HEVC) standard in 2013 [2]. This standard is able to reduce the bit rate by 50% compared with its predecessor, namely H.264/*Advanced Video Coding* (AVC) [3], while maintaining the same objective quality [4]. Beyond HEVC, the *Versatile Video Coding* (VVC) standard [5], which has been developed by the *Joint Video Experts Team* (JVET), has emerged strongly, while the *Alliance for Open Media* (AOM) [6], a joint development foundation, is targeting the *AOMedia Video 1* (AV1) codec as a royalty-free video coding format. However, since both VVC and AV1 massively increase coding complexity with regard to their predecessors, it is obvious that most traditional coding techniques have been exhausted, and therefore new alternatives need to be explored.

Perceptual video coding using computational models of visual attention has recently been recognized as a promising approach to providing a new pathway for additional video compression based on human visual characteristics. The idea behind most of the existing visual attention-based video coding methods is to encode a small area around the gaze locations using a higher quality compared with other less visually important regions. Such spatial prioritization is supported by the fact that only a small region of several degrees of the visual angle around the center of gaze is perceived with high spatial resolution. This is due to the highly non-uniform distribution of photoreceptors on the human retina [7]. Thus, perceptual video coding tries to achieve greater compression in those areas of the frame that do not receive the attention of the viewer, while the quality of the areas to which the user pays more attention is preserved. By doing this, even though the objective quality would be, of course, lower, there would not be a significant impact on the subjective quality perceived by the viewer.

In the literature, several computational models of visual attention have been developed to predict gaze locations in digital images and video [8]. Although the current visual attention models provide an easy and cost-effective way for gaze prediction, they are still imperfect. One must consider that human attention prediction is still an open and challenging problem. Ideally, the most accurate approach to finding actual gaze locations is to use an eye-tracking device. In a typical eye-tracking session, the gaze locations of a human observer are recorded when watching a given video clip using a remote screen-mounted or head-mounted eye-tracking system. Eye tracking technology [9] allows you to know, by means of different sensors and/or cameras, the point on the screen which the viewer is looking at with a very high degree of precision.

In this paper, eye tracking technology will be used to determine the parts of a screen that is being recorded that should be compressed more aggressively (since the viewer pays less attention to them), and the parts that should be compressed less. To highlight the importance of this proposal, we can see how many teachers and professors have been recording their classes during the COVID-19 pandemic. This has generated a great number of screen-recorded videos which could have been benefited from this proposal. In fact, it is expected that teaching change a lot after this experience and many teachers and professor may start recording more and more classes in front of a computer and it is essential to reduce the size of the videos that are produced.

The main contribution of this work is to present a system that makes use of eye tracking data to obtain additional bit rate reduction. The proposed system makes use of video recorded from the scene camera of the eye tracking glasses and an HEVC encoder using real gaze locations for video compression. The results of encoding several test sequences and showing them to several viewers indicate that the proposed system obtains a bit rate reduction of up to 31.3% compared with a standard HEVC encoder, while the subjective quality is preserved.

The remainder of this paper is organized as follows. Section 2 describes the technical background of the HEVC standard, and the related work. Section 3 introduces the proposed algorithm, and the experimental results are given in Section 4. Finally, Section 5 concludes the paper.

## Technical Background and Related Work

### Technical Background

The main difference of HEVC with respect to H.264/AVC is the picture partitioning: while H.264/AVC used the traditional approach based on *Macro-Blocks* (MBs) for the *Motion Estimation* (ME) and *Blocks* for the transform, HEVC defines four new concepts: *Coding Tree Unit* (CTU), *Coding Unit* (CU), *Prediction Unit* (PU), and *Transform Unit* (TU).

Each picture is partitioned into square regions of fixed size called CTUs. Then a quadtree structure is applied to each CTU, dividing it recursively into CUs with a size that can vary from 64$\times$64 to 8$\times$8 pixels. Each of these CUs may contain one or more PUs (which are the units where predictions are carried out) and one or more TUs (which are the units where the transform is applied).

As in previous standards, a *quantization parameter* (QP) is present in the configuration of the video stream. This QP ranges from 0 to 51, and a variation of six units means that the quantization factor for the quantization matrix is doubled. In HEVC, the encoder can signal whether or not to use quantization matrices enabling frequency dependent scaling. Frequency dependent scaling is useful when carrying out human visual system (HVS)-based quantization, where low frequency coefficients are quantized with a finer quantization step size when compared with high frequency coefficients in the transform block. In HEVC, an initial QP value for every slice is coded in the *Picture Parameter Set*, which corresponds to the QP of the first CU in the slice. Then, the differential QP value with respect to the previous one is encoded in each CU. The QP value may need to be changed within a picture, for example for rate control and perceptual quantization purposes.

### Related Work

Obtaining the region of interest of an image is not a new topic in computer vision, and it has attracted the attention of many researchers. For instance, the authors in [10] propose a method based on maximizing the information from the frame by using Shannon’s self-information measure and neural networks. Neural networks are used since they have also been shown to mirror the behavior and the neuronal architecture of the early primate visual system [8]. In fact, with the evolution of neural networks, they have been more and more used for this purpose and, for instance, Generative Adversarial Networks (GANs) are used in [11] to color salciency maps.

Some works focus on extracting the saliency areas from encoded videos. In the framework of an H.264/AVC encoded sequence, in [12] the authors present, compressed-domain features based on the study of visual attention in humans. The first one is the *Motion Vector Entropy*, which is an quantitative measurement of MV variability. The other metric defined in the work is the *Smoothed Residual Norm*, which involves the application of a smoothing filter on the the norm of the quantized transformed prediction residual of an MB. After performing a series of tests, the results confirm that they can be used to describe a simple saliency estimation without fully reconstructing the video.

Regarding the combination of saliency maps with quantization in rate-distortion optimization, different modes may obtain a different video quality and bit rate. The mode decision is usually determined by minimizing the cost function (encoding error plus bit rate multiplied by a Lagrange multiplier). Considering that the Lagrange multiplier will affect the mode decision in rate-distortion optimization, a Lagrange multiplier adjustment method is explored in [13]. An optimized rate control algorithm with foveated video is proposed in [14], and the foveal peak signal-to-noise ratio (FPSNR) is introduced as a means of subjective quality assessment.

The authors in [15] propose the computation of a saliency map for every frame and then the QP is varied according to the saliency of a given MB and the QP of the collocated MB in the previous frame. The authors report a bit rate saving of 26%. Similarly, authors in [16] propose a technique that is able to reduce the bit rate by 26% on average with respect to the *x264* encoder in which one user needs to use an eye-tracking system to watch the sequence, from which a multi-user saliency map is derived.

A similar approach to obtain saliency maps, but in this case using features of HEVC-encoded videos, is presented in [17]. Nevertheless, in this proposal the final objective is not to reduce the bit rate of the encoded video, but only to predict the saliency areas. Moreover, a dataset with fixation points is provided with the publication and this dataset shows that some sequences present more than one fixation point in a single frame. However, in the scenario of reducing the bit rate of a sequence, having more than one fixation point will degrade the performance, since it will imply many variations in the QP in a single frame and, therefore, encoding more variations of this parameter in the bit stream. Moreover, it would also imply a too large area encoded with a high QP value, minimizing the bit rate reduction and, in the worst case, combined with the QP variations, might even produce a greater bit rate than the one of the original sequence. Therefore, methods which only consider one fixation point per sequence would be the best.

Regarding HEVC as well, but with the objective of achieving a bit rate reduction, a saliency-based QP modification method is proposed in [18]. As in the previous cases, the authors first compute a saliency map and, after that, the QP of a given block is modified on the basis of the mean saliency of the pixels that compose it and the mean saliency of the frame. The results show that they are able to reduce the bit rate by 12.1%, 9.1%, 7.2%, and 6.6% for QP values of 22, 27, 32, and 37, respectively, with a negligible impact on subjective quality. More recently, authors in [19] measure the saliency of each CTU using the luma texture. However, the objective of this work is not to reduce the needed bit rate, but to increase the subjective quality of the video by decreasing the QP of the CTUs to which viewers tend to pay more attention. A similar approach to increase the subjective quality without increasing the bit rate is presented in [20]. For that purpose, authors combine three techniques: static saliency detection, dynamic saliency detection, and adaptive bit rate allocation.

## Proposed Dynamic Perceptual Quantization Algorithm

One of the disadvantages of the above works, such as [15, 16] and [18], is that they have to compute the saliency maps of every frame, what requires an overhead in encoding time. However, nowadays it is possible to follow the position on the screen where an eye is looking. Moreover, eye tracking technology is also included in devices such as smartphones, with the aim, for instance, of authentication [21]. This technology has even been perfected along the years with algorithms that, for instance, detect possible drifts in the original position of the eye-tracking device [22].

Furthermore, we can assume that if a person looks at a specific point in a frame, it means that something is catching their attention at that point and it is very likely that a different person will also look at that same point in the future if the same sequence is played.

Taking into account that the scenario of the proposal is a screen that is being recorded, a *Dynamic Perceptual Quantization Algorithm* (DPQA) is proposed by using the fixation points that are catching the attention of a viewer. Then, these points are used to modify the QP value in the neighboring area.

It can be seen that if a viewer needs to be watching the screen, the change cannot be applied to the current frame, but to the one which is going to be encoded after it. However, given the temporal proximity, the viewer is not expected to change the fixation point significantly and the fixation point of a frame can be interpolated to the following ones.

Regarding complexity, while computing the saliency maps requires $\mathcal {O}(n)$ operations for each frame, *n* being the number pixels, the proposed methods only requires $\mathcal {O}(1)$ operations for each one.

### Quantization Levels

The principal problem is the definition of the area that will be affected by the change in the quantization. Even though the QP value may change for each CU, as stated in Section 2, it must be considered that a QP changing too frequently will lead to a bit rate increment since the QP value is encoded in a differential way. For this reason, the CTU is the basic unit that has been chosen for QP change in our algorithm.

**Figure 1:**
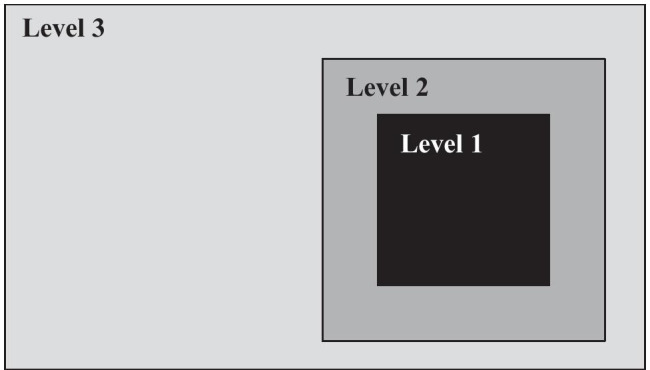
Division of the frame in 3 levels of quantization.

The frame is divided into 3 quantization levels, as shown in Fig. 1. The first level corresponds to the area in which the viewer focuses their attention, i.e. a high attention area. The second level is the area surrounding the first level and is considered as a medium attention area. Finally, the third level is a low attention area, containing the rest of the frame.

Areas corresponding to levels 1 and 2 are composed of an odd number of CTUs, the CTU being that which contains the point which the spectator was looking at in that frame. Consequently, these levels are concentric, while level 3 is not. The reason that the areas are rectangles instead of circles is the same as that for the basic unit for QP changing being the CTU instead of the CU: to prevent quick switching between QP values.

The QP value for level 1 is always the base QP used for that slice, $QP_{base}$, while the QP value for levels 2 and 3 are $QP_{base}+4$ and $QP_{base}+8$, respectively. This results in multiplying the quantization factor by, approximately, 1.5 and 2.5, respectively.

### Sizes of the Areas for the Levels of Quantization

In the proposed algorithm, the areas that corresponds to levels 1 and 2 are specified as a percentage of the frame. The area of the sum of levels 1 and 2 has been defined to be 75% of the whole frame. Additionally, the area corresponding to level 1 is dynamically adapted by taking into account the variance of the fixation point in the last frames. If the variance is high, then it means that the viewer is moving their eyes and, therefore, the area that is not affected by an increment in the QP value should be bigger. Otherwise, if the variance is low, it means that the viewer is fixing their gaze on a specific location and, therefore, the area that is not affected by an increment in the QP value should be smaller. Hence, the area of level 1 has been defined to be 20%, 30%, or 40% of the whole frame depending on the variation of the fixation point. In order to calculate this variance, it must be considered that the fixation point consists of a 2-dimensional variable, $\mathbf{p} =(x,y)$. The final variance has been considered as the infinity norm of the variances of each coordinate, as defined in (1), for the last 10 frames.

1 $$\begin{aligned} \left\| \mathrm {Var}_p \right\| _{\infty } = \max \left( \mathrm {Var}_x, \mathrm {Var}_y \right) \end{aligned}$$

In (1), $\mathrm {Var}_x$ and $\mathrm {Var}_y$ denote the variance of the coordinates *x* and *y*, respectively, of the fixation point of the viewer for the last 10 frames.

The infinite norm has been chosen instead of other usual norms, such as the 2-norm (Euclidean distance), since this is the norm that gives the maximum value out of all the usual ones. This means that the area of level 1 increases when the variation of the movement occurs in any direction, either vertically or horizontally.

### Variance Threshold for Level 1 Area

**Figure 2:**
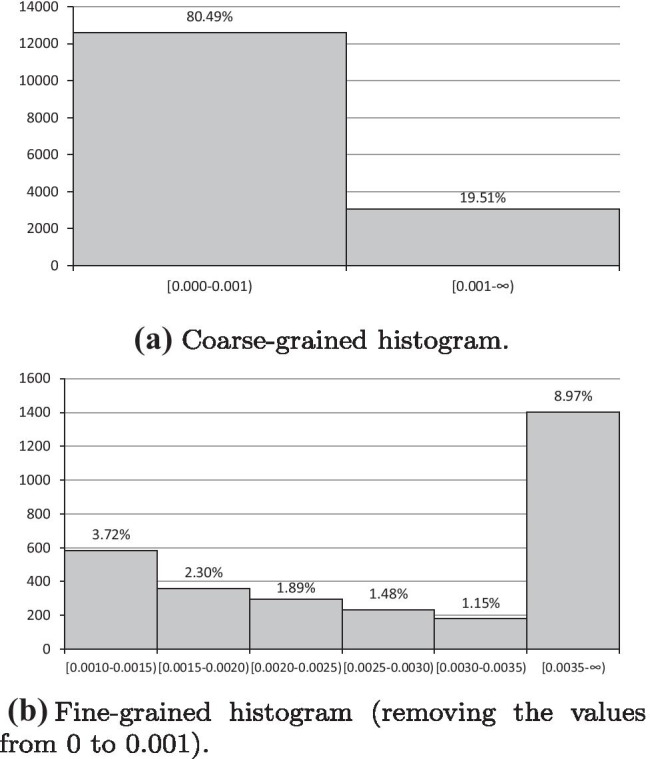
Histogram of the distribution of the $\left\| \mathrm {Var}_p \right\| _{\infty }$ variable.

In order to set the threshold of $\left\| \mathrm {Var}_p \right\| _{\infty }$ for which the area of level 1 is switched from one value to another, a study of the value of the variance was carried out. The variance was calculated for all the frames and all the sequences in the document of the *Common Test Conditions* published by the JCT-VC [23]. Thus, after measuring the relative fixation points of a viewer (i.e., the top-left corner is the position (0, 0) and the bottom-right corner is (1, 1)), the histogram of the variable can be seen in Fig. 2.

From Fig. 2a, it can be concluded that in 80% of the cases the variance of the last 10 frames falls below the value of 0.001, what supports the idea that of extrapolating the fixation points. Furthermore, because of this, the threshold for switching the area of the first level from 20% to 30% has been set to 0.001.

Regarding the second threshold, when focusing on the second histogram (Fig. 2b), which removes the values from 0 to 0.001, it can be seen that 4% of the values fall below 0.0015 and, then, the density becomes lower and lower. Because of this, the threshold has been set to 0.0015. Therefore, the area of the first level of quantization if given by (2).

2 $$\begin{aligned} \small \mathrm {L1} \; \mathrm {Area} \; (\%) = \left\{ \begin{array}{lcc} 20\% &{} \mathrm {if} &{} \left\| \mathrm {Var}_p \right\| _{\infty } \le 0.001 \\ \\ 30\% &{} \mathrm {if} &{} 0.001 < \left\| \mathrm {Var}_p \right\| _{\infty } \le 0.0015 \\ \\ 40\% &{} \mathrm {if} &{} \left\| \mathrm {Var}_p \right\| _{\infty } > 0.0015 \end{array} \right. \end{aligned}$$

In accordance with this, and considering that the sum of the areas of levels 1 and 2 must always be 75%, the remaining area for level 2 is 55%, 45%, or 35% of the frame, depending on the value of $\left\| \mathrm {Var}_p \right\| _{\infty }$.

### Actual Regions for Each Level of Quantization

As has been stated above, the modification of the QP value is performed on a CTU basis. Because of this fact, the percentages of the areas shown above are translated into an integer number of CTUs. As the CTU that contains the fixation point should be the one at the center of the regions, an odd number of CTUs is always chosen. The number of CTUs in each direction, horizontally and vertically, is derived as shown in (3).

3 $$\begin{aligned} \#({\mathrm{CTU}}_H) &{}= \left\lfloor \sqrt{\frac{{\mathrm{Area}} \; (\%)}{100}} \left\lceil \frac{w}{64} \right\rceil \right\rfloor + \delta _H \\ \\ \#(\mathrm {CTU}_V) &{}= \left\lfloor \sqrt{\frac{{\mathrm{Area}} \; (\%)}{100}} \left\lceil \frac{h}{64} \right\rceil \right\rfloor + \delta _V \end{aligned}$$

Here, *w* and *h* represent the width and the height of the frame in pixels, and the ceiling of that value divided by 64 gives the number of CTUs in each direction. This number of CTUs is multiplied by the squared root (which is used to split the two components, horizontal and vertical) of the proportion of the area. Also, $\delta _H$ and $\delta _V$ may be 0, if the first term of the summation is odd, or 1 otherwise. With these terms the we ensure that the resulting number of CTUs is odd.

Therefore, the CTU containing the fixation point will be the center of the area, which will expand $(\#(\mathrm {CTU}_H) - 1)/2$ CTUs to the left and to the right, and $(\#(\mathrm {CTU}_V) - 1)/2$ to the top and to the bottom of the frame. If, in any direction, there are not enough CTUs, the area is not displaced, but the remaining CTUs are subtracted from the number calculated above.

For instance, if a Full HD video (30 $\times$ 17 CTUs) is considered with a level 1 area of 20%, we obtain $\#(\mathrm {CTU}_H) = 13$ and $\#(\mathrm {CTU}_V) = 7$. Therefore, if a viewer fixes their attention at the point (1700, 600), which belongs to the CTU located at (26, 9), the area will expand as a rectangle from the CTU (20, 6) to the CTU (29, 12). It is easy to see that, in this case, there are not enough CTUs on the right of the fixation point, so the final rectangle is composed of 10 $\times$ 7 CTUs, instead of 13 $\times$ 7. Thus, the actual proportion of the area of the frame is roughly 14%.

### Overall Frame Processing Algorithm

**Figure 3:**
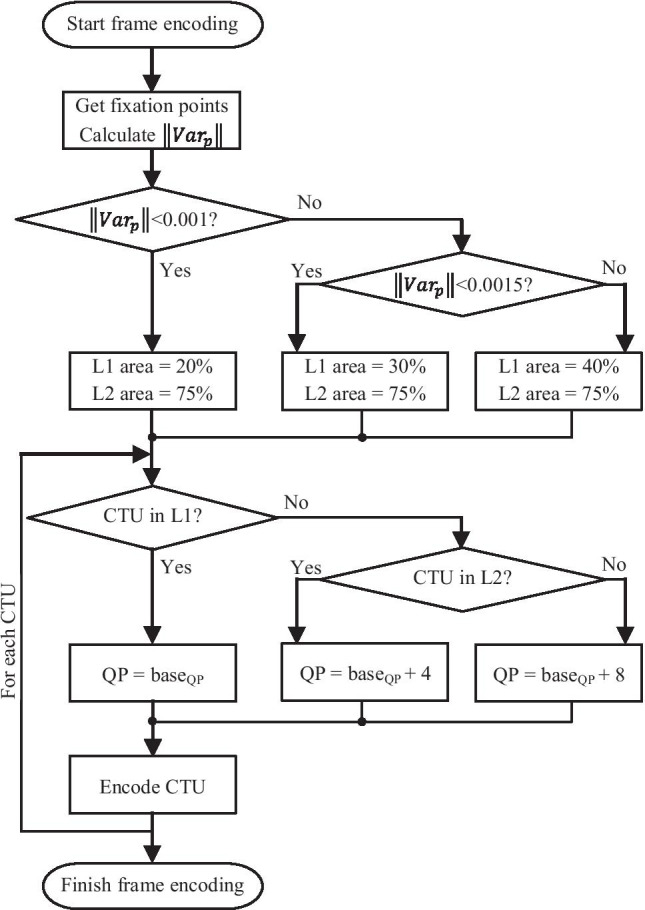
Proposed QP variation algorithm.

To finish the section, Fig. 3 shows a diagram of the proposal for frame processing as described in the previous subsections. In this figure, you can see the decision on the area of level 1 according to the variance of the fixation point, and the QP selection for each CTU based on the area in which it is contained.

## Performance Evaluation

### System Setup

In this work we use a head-mounted eye tracking system for tracking the eye movements based on the video-based combined pupil and corneal reflection measurement methodology. A binocular version has been chosen to capture the movement of both pupils, with two cameras focused towards the corresponding eye. A third camera, called the *world camera*, is responsible for capturing everything that encompasses the field of vision.

These cameras together with an image processing software are used to track the head position relative to the eyes. Cameras that detect pupil movement can be adjusted by rotation and sliding to adapt their position and correctly detect the eyeball. These cameras can also be configured in various resolutions with several associated frame rates.

In order to test the proposal, the different sequences considered in [23] were encoded in HEVC with the reference software HM 16.6 [24] and with the same software but modified to include the proposed algorithm. The encoding configuration was set to *Low Delay P* (LP), as defined in [23]. This configuration was chosen given that, as the scenario is a screen being recorded, it does not make sense to use a configuration in which the encoding order is not the same as the presentation order, such as *Random Access* (RA). Furthermore, the sequences were encoded using base QP values of 22, 27, 32, and 37, as specified by the document.

### Test Material and Metrics

The HEVC common test conditions [23] define a set of test video sequences with different characteristics. The sequences used in this performance evaluation were those proposed for the LP configuration in [23], grouped by classes according to their resolution (class A was not used since, according to the test conditions, it should only be used with the RA configuration):

- Class B (1920x1800 pixels): *BasketballDrive*, *BQTerrace*, *Cactus*, *Kimono*, and *ParkScene*.
- Class C (832x480 pixels): *BasketballDrill*, *BQMall*, *PartyScene*, and *RaceHorsesC*.
- Class D (416x240 pixels): *BasketballPass*, *BlowingBubbles*, *BQSquare*, and *RaceHorses*.
- Class E (1280x720 pixels): *FourPeople*, *Johnny*, and *KristenAndSara*.

Regarding the metric used during the tests, a subjective quality metric was chosen, given the nature of the problem. It is clear that the algorithm will perform worse in terms of rate-distortion, as shown in Table 1 by the use of the BD-rate metric [25], which measures the increment in the bit rate to keep the same *Peak Signal-to-Noise Ratio* (PSNR). It shows an increment of 5.2% on average with respect to the original encoded sequence. However, the objective in this work is to demonstrate that the bit rate can be reduced preserving the same subjective quality.

**Table 1:** BD-rate (%) comparison of the proposed DPQA algorithm.

		BD-rate (%)
Class B	BasketballDrive	4.6
	BQTerrace	8.9
	Cactus	3.3
	Kimono	5.2
	ParkScene	9.6
Class C	BasketballDrill	0.5
	BQMall	5.1
	PartyScene	9.1
	RaceHorsesC	5.8
Class D	BasketballPass	1.8
	BlowingBubbles	11.1
	BQSquare	8.3
	RaceHorses	3.7
Class E	FourPeople	1.1
	Johnny	4.1
	KristenAndSara	1.0
Average		5.2

**Table 2:** MOS results when comparing the original sequence with itself and the original with the one encoded with the proposed algorithm (*QP*_*base*_ = *27*).

		Original vs. itself		Original vs. prop.	
		Avg.	CI	Avg.	CI
B	BasketballDrive	4.89	[4.80-4.98]	4.72	[4.54-4.90]
	BQTerrace	4.75	[5.59-4.91]	4.78	[5.63-4.92]
	Cactus	4.64	[4.50-4.78]	4.86	[4.74-4.98]
	Kimono	4.67	[4.53-4.81]	4.72	[4.58-4.87]
	ParkScene	4.61	[4.45-4.78]	4.83	[4.73-4.93]
C	BasketballDrill	4.58	[4.42-4.74]	4.67	[4.53-4.81]
	BQMall	4.56	[4.37-4.74]	4.64	[4.47-4.80]
	PartyScene	4.78	[4.62-4.64]	4.72	[4.56-4.88]
	RaceHorsesC	4.69	[4.55-4.84]	4.72	[4.60-4.85]
D	BasketballPass	4.22	[3.96-4.48]	4.00	[3.71-4.29]
	BlowingBubbles	4.50	[4.33-4.67]	4.31	[4.10-4.51]
	BQSquare	4.44	[4.26-4.63]	4.31	[4.07-4.54]
	RaceHorses	4.58	[4.44-4.73]	4.31	[4.10-4.51]
E	FourPeople	4.72	[4.58-4.87]	4.69	[4.54-4.85]
	Johnny	4.64	[4.50-4.78]	4.72	[4.23-4.65]
	KristenAndSara	4.89	[4.80-4.98]	4.61	[4.43-4.79]
Average		4.64	[4.60-4.68]	4.60	[4.55-4.65]

**Table 3:** Bit rate saving (%) when comparing the original sequence with the one encoded with the proposed algorithm.

		QP=22	QP=27	QP=32	QP=37
B	BasketballDrive	24.0	13.8	9.9	8.8
	BQTerrace	31.3	24.1	13.7	10.3
	Cactus	26.3	14.2	10.1	8.6
	Kimono	17.0	13.8	10.7	10.7
	ParkScene	21.0	16.5	13.5	12.3
C	BasketballDrill	15.4	12.9	10.3	9.3
	BQMall	16.6	12.5	9.9	8.5
	PartyScene	20.9	16.7	14.6	13.3
	RaceHorsesC	19.5	14.6	11.8	10.7
D	BasketballPass	17.5	15.0	12.4	11.0
	BlowingBubbles	19.8	16.9	15.6	10.3
	BQSquare	22.5	16.2	13.9	13.3
	RaceHorses	14.6	11.9	9.9	8.3
E	FourPeople	17.5	10.2	7.3	6.4
	Johnny	23.1	10.7	4.3	3.7
	KristenAndSara	21.9	13.4	8.9	6.3
Average		20.6	14.6	11.1	9.5

Therefore, among all the subjective quality metrics, the *Mean Opinion Score* (MOS), defined in the ITU-T P.910 recommendation about subjective video quality assessment methods [26], was chosen since it is the one used in most of the perceptual experiments in the literature. Regarding the methodology, the *Degradation Category Rating* (DCR) methodology [26] was chosen because the objective is to compare two given sequences since, as pointed out in the ITU-T P.910 recommendation, DCR is the method suitable when it is important to compare the fidelity of a signal with respect to the source signal, which is the case of this study.

In the DCR method, the viewers are shown the sequences in pairs, where the first stimulus is the reference sequence without modifications and the second one is the sequence with modifications. Between the first and the second stimulus 2 seconds of gray video are played to ‘refresh the eye’. In addition 10 seconds of gray are also played between pairs to give time for the viewer to give a score. The viewer is asked to score each pair with an integer number by answering the question: “How much degradation from the first to the second sequence do you perceive?” After that, they need to assess the degradation in a scale from 9 (which means that the degradation is imperceptible) to 1 (which means that the degradation is very annoying). Finally, this scale is re-scaled to one from 5 to 1 to comply with the standard according to Equation (4).

4 $$\begin{aligned} MOS = \left\lfloor \frac{score+1}{2} \right\rfloor \end{aligned}$$

The experiment was conducted for $QP_{base}=27$ and with 18 people who did not have previous experience with this kind of tests. They were divided into groups of 3 or 4 people so that they could have a good angle of vision. A 58-inches screen with Full HD resolution (according to the maximum resolution of the sequences that were tested) was used to during the assessments. The viewers were located about 2 meters away from the screen and with a space between them that ensured that they could not interact nor see the assessments of other participants, but at the same way their angle with respect to the screen was small so that all of them had a similar perception.

As the experiment is only conducted with a sample of the population, instead of just calculating the average of the scores, it is usual to also calculate a Confidence Interval (CI) for the mean of the population: in this case, a 90% confidence interval in which any assumption about the population is made.

Furthermore, in an attempt to detect the presence of autosuggestion, the experiments were carried out twice, telling the participants that they were going to score different proposals. However, in one of them, both stimuli corresponded to the original sequence, what can be used to produce a control score.

### Results

The results of the MOS metric for $QP_{base}=27$ are shown in Table 2. If the viewers do not autosuggest, the MOS result of comparing a sequence with itself should be 5. However, it can be seen that the results in both cases are very similar (and very close to 5). In order to check whether there is a significant difference in the scores given to the sequences by the participants, a *Mann-–Whitney U test* [27] was conducted (since the populations do not follow a normal distribution). The null hypothesis, $H_0$, is that the populations follow the same distribution (i.e., they have an identical mean), while the alternative hypothesis, $H_a$, is the opposite. After executing the test, the p-value is 0.762, which means that for any acceptable level of significance, $H_0$ cannot be rejected and, therefore, it can be considered that the mean score of the sequences is the same when showing a sequence encoded with the proposed algorithm and when showing the original sequence.

**Figure 4:**
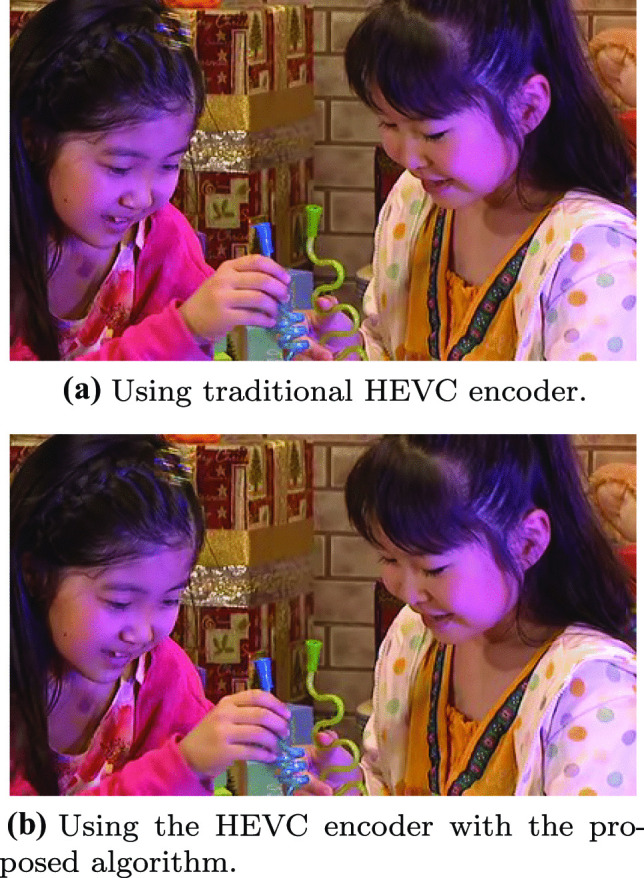
Visual comparison of the fith frame of sequence BlowingBubbles using the traditional HEVC encoder and the HEVC encoder implementing the DPQA algorithm.

Figure 4 shows the fifth frame of sequence *BlowingBubbles* (Class D, 416x240 pixels) encoded with the original HEVC encoder (Fig. 4a) and with the encoder using the proposed DPQA algorithm (Fig. 4b). As it can be seen, there is not visual difference between them, what includes the absence of blocking effects in the edge between different levels of quantization.

Moreover, Table 3 shows the results for bit rate saving with QP values 22, 27, 32, and 37. It can be seen that the proposed algorithm achieves an average bit rate reduction of 20.6%, 14.6%, 11.1%, and 9.5%, respectively. Furthermore, if this is compared with encoding the same video with H.264/AVC, a total bit rate reduction of 57% can be reached by using HEVC with the proposed algorithm.

Finally, when comparing the proposal with other state-of-the-art algorithms, the most similar one for HEVC is [18], in which the authors report a bit rate saving of 12.1%, 9.1%, 7.2%, and 6.6% for the same QP values, being outperformed by the proposed DPQA algorithm by a 60% of bit rate saving on average without any impact in the perceived quality either. If it is compared with [15], in that work, the authors report a 26% of bit rate saving for H.264/AVC-encoded videos. Therefore, when comparing the savings of the proposed algorithm with respect to H.264/AVC (57% on average), it obtains greater savings than that algorithm.

## Conclusions and Future Work

In this paper, we present a new algorithm for bit rate reduction of screen recorded sequences based on the visual perception of videos. An eye tracking system is used during the recording to locate the fixation point of the viewer. Then, the area around that point is encoded with the base QP value, which increases when moving away from it.

A total of three different levels of quantization are used. The area corresponding to the first level (the area of greatest attention) is dynamically adapted according to the variation of the fixation point of the user in the last frames.

The results show that the perceived quality is not affected when compared with the original HEVC-encoded sequence, while the bit rate can be reduced by 21% when using a QP value of 22.

Regarding the future works to be done in the topic, a machine learning algorithm can be considered to try to predict the interest point (or points if there are several). This approach would help to eliminate the need of an eye tracker what, at the same time, would make the proposal valid for a wider range of scenarios.
